# Determination of thoracic and lumbar spinal processes by their percentage position between C7 and the PSIS level

**DOI:** 10.1186/1756-0500-6-58

**Published:** 2013-02-11

**Authors:** Markus J Ernst, Fabian M Rast, Christoph M Bauer, Valentine L Marcar, Jan Kool

**Affiliations:** 1 Zurich University of Applied Sciences, Institute of Physiotherapy, Research & Development, Technikumstrasse 71, 8401, Winterthur, Switzerland

**Keywords:** Manual palpation, Lumbar, Thoracic, Spinal process, Percentage position, Reliability, Flexible ruler

## Abstract

**Background:**

Accurate measurements of spinal movement require reliable determination of anatomical landmarks. Current methods of identifying these are not sufficiently reliable or valid for this purpose. A reliable and convenient method of placing markers on selected vertebra is needed to compare measurements between different testers, subjects and sessions.

**Findings:**

Two testers palpated T4, T7, T10, L1 and L4 spinal processes according to established criteria. They measured the position of spinal processes between C7 and the Posterior Superior Iliac Spine (PSIS) at the Pelvis independently using a flexible ruler placed on the spine. Subjects with a wide range of body heights but without visible spinal deformities were recruited for measurements. Reliability was calculated using absolute and relative values. Mean percentage position and 95% Confidence Intervals were calculated using the mean of both testers’ measurement for all subjects.

Twenty-two subjects participated. The mean distance between C7 and the PSIS level was 50.9 cm (SD: 3.5 cm). Relative reliability for all spinal processes was almost perfect (ICC: > 0.9). Absolute reliability values showed high agreement between testers. Percentage position of T4 was found to be situated 21% along the distance between C7 and the PSIS level, T7 at 39%, T10 at 54.1%, L1 at 70.9% and L4 at 86.1% accordingly. 95% Confidence intervals around mean percentage positions had a maximum at L1 with 2.8% range from upper to lower limit.

**Conclusions:**

The distance of three thoracic and two lumbar spinal processes can be reliably and accurately measured by independent testers, using a flexible ruler. Percentage positions between C7 and PSIS level correspond to spinal processes for subjects without visible deformities in the sagittal and frontal plane.

## Findings

### Background

Movement, captured by high frequency opto-electronic cameras is considered the most accurate and reliable method for measuring trunk and limb movements in a laboratory setting [1,2]. To define movement segments in the lab, (for example the lumbar or thoracic spine), researchers use kinematic models. These models require markers or sensors to be placed on anatomical landmarks, such as spinal processes. Authors may state explicitly which vertebrae were used [3-5], but frequently fail to describe the palpation method used to identify a particular vertebra or the reliability and validity of the approach taken. Determining specific spinal processes is difficult which has led to the reliability and validity of identifying a spinal process being questioned [6-10]. However, if a marker/sensor is placed above or below the intended location, the results of a kinematic model may be erroneous.

As part of a research procedure studying the validity of different movement tests of the trunk, lumbar spine and adjacent regions, we needed a reliable and convenient method for repeatedly placing markers and sensors on the same location of the back of a subject when the subject is in the upright standing position [11]. The use of anatomical landmarks and the assessment of behavioural properties (e.g. the spinal process C7 does not move forward, during neck extension but C6 does) greatly enhances the accuracy in placing a marker/sensor on the intended vertebra or very close to it [12].

Flexible rulers have been used as a tool in clinical measurements, to determine body postures like kyphosis and pelvic alignment. Their validity and reliability has been controversially reported [13]. In a recent study by Dunleavy et al. a flexible ruler was used to measure spinal length parameters, which were subsequently traced onto paper [14]. In measurements of the length and curvature of the spine, these authors found moderate to high intra-and moderate inter-tester reliability (SE measurement: 0.58 - 2 cm). The measurement error in the lumbar spine (2 cm) corresponds to 17% of the lumbar spine length. The main source of error as identified by the authors is the removal of the flexible ruler from the back. We therefore decided to perform all measurement directly on the back in order to circumvent this source of error.

The aims of the current study are:

To examine the inter-tester reliability in measuring the distance between palpated spinal process of the thoracic and lumbar spine, using a flexible ruler, and measuring directly on the spine.

To determine the percentage position of 3 thoracic and 2 lumbar spinal processes on the distance between C7 and the PSIS level.

## Methods

Twenty-two healthy adults (11 female and 11 male), Caucasians of slender type with no visible greater deviations in the frontal (scoliosis) or sagittal plane (kyphosis or hyperlordosis), as judged by an experienced physiotherapists, were included in the study. Subjects were excluded if they had any diagnosis of specific or chronic non-specific back pain conditions, changed morphology of the spine, or were not able to stand upright for about half an hour. The study involved six testers who performed measurements in pairs. Pairs of testers were composed according to availability. Testers were either movement scientists or physiotherapists employed at the Institute of Physiotherapy at the Zurich University of Applied Science and had a working experience between 1 and 18 years. The number of measurements performed by a tester varied between 2 and 15. Testers collaboratively determined C7, T4, T7, T10, L1, L4 and the PSIS on both sides. Measurements took between 25 and 45 minutes.

During a measurement subjects stood in an upright position with feet hip width apart. During periodic breaks subjects were able to change this position, but were required to return to it when measurements continued. Testers adhered to the palpatory standards described by Fields 2006 and others [15-24].

We differentiated between spinal process C7 and C6 by the fact the former does not move forward, when the neck moves in extension, but the latter does. Its location was marked.

Left and right PSISs are located at the height of S1 or S2 and were palpated from below and their location marked.

**Figure 1:**
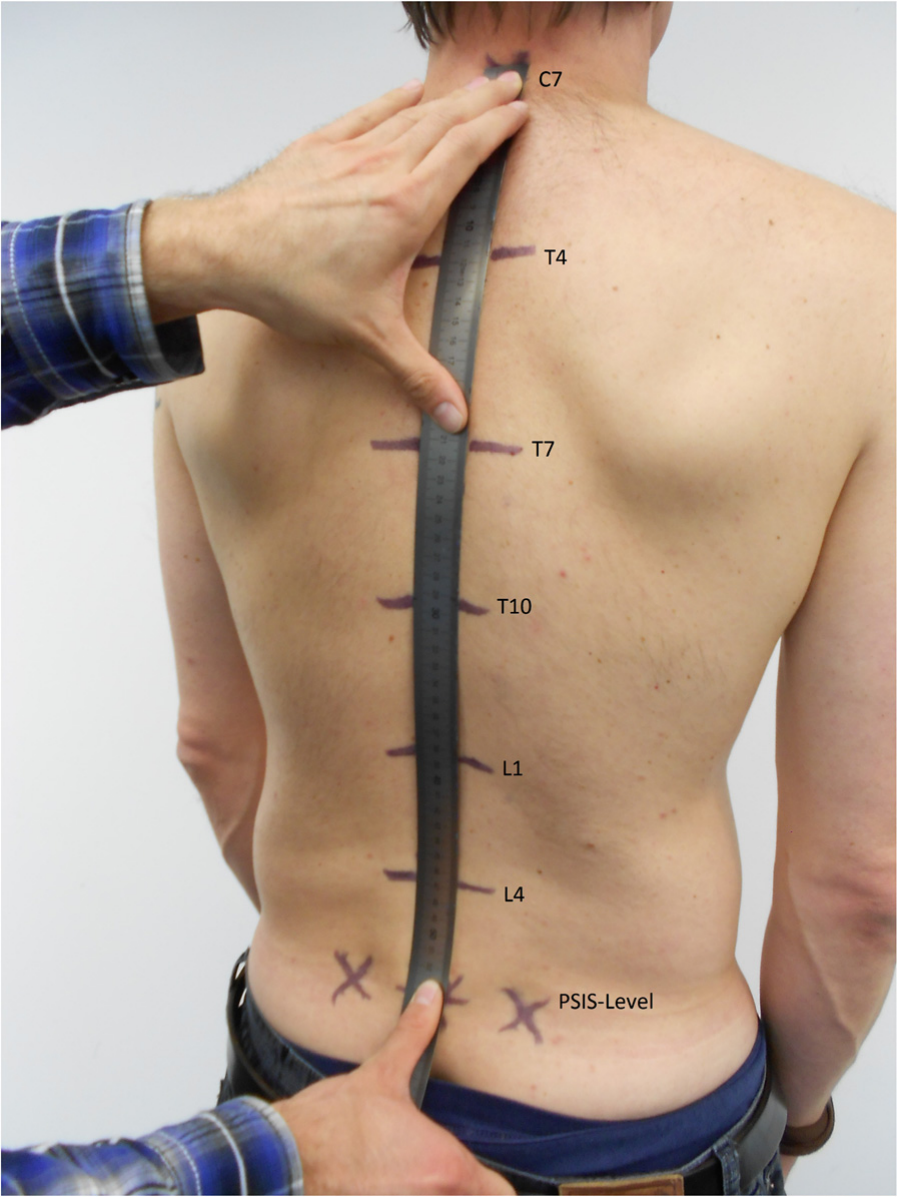
Flexible Ruler placed on the back with selected spinal processes in an exaggerated manner for illustration purposes.

The flexible ruler, length 100 cm was placed on the spine so that it followed its contour and connected C7 and PSIS. The position of T4, T7, T10, L1, L4 along the ruler was recorded in cm (Figure 1).

T4 was located by counting down vertebrae from C7.

Reports by Field 2006 that T7 is located at the height of the lower scapula tip, has recently been questioned suggesting instead, that T9 is situated at this location [16,17]. We identified T7 by counting down vertebrae starting at T4; and incidentally confirmed T9 to be located at the lower scapular tip more often than not.

T10 was located by counting down vertebrae starting from T7.

L1 was located by counting down vertebrae from T10 and confirmed by its more rectangular shape and increased height as compared to T12, which is more rounded [10].

L4 was located by counting down vertebrae from L1 and confirmed by counting up vertebrae from PSIS level. The identification was aided by it having a larger spinal process and a rectangular shape compared to the smaller and deeper L5 [18]. As a final check we confirmed its approximate position corresponded to the iliac crest level [19,20]. The most dorsal point of a selected spinal process was identified independently by each tester. Its ultimate location was reached by mutual consent between the testers involved and marked. When agreement was reached on a specific vertebra, one tester measured and recorded its distance to C7, while the other faced away. This was followed by the other tester performing the same measurement. For each vertebra we therefore had two independent and blinded distance measurements (Figure 1). Care was taken that the ruler did not slide over the skin when a measurement was made. Each tester independently checked the positions of their previously measured vertebrae along the ruler and that the total length between C7 and PSIS level was correct. In addition to the absolute position, we calculated the relative percentage position of each vertebra by dividing its distance from C7 by the length between C7 and PSIS and multiplying this value by 100.

### Ethics

The Study has been approved by the ethical committee of the Canton Zurich Switzerland (KEK-StV-Nr.20/10). Written informed consent was obtained from all participants.

### Data analysis

Intertester-reliability for the distance C7 to the PSIS level, and the position of T4, T7, T10, L1 and L4 spinal process were examined using Bland and Altman’s Limits of agreement for absolute values. The Intraclass Correlation Coefficient (ICC Model 1, 1) was used to assess relative reliability [25]. Landis and Koch consider reliability values below 0.2 as slight, between 0.21 and 0.4 as fair, between 0.41 and 0.6 as moderate, between 0.61 to 0.8 as substantial and with values above 0.81 as almost perfect [26]. The Standard Error of the measurement (SEM) estimates the spread in measurements in absolute values [12,26,27]. Mean percentage position of each vertebrae and its 95% Confidence interval were calculated from the mean position recorded by both testers. Metric results are given with an accuracy of one decimal place. Data analysis was conducted using R, a language and environment for statistical computing (http://www.r-project.org) Additional file 1.

## Results

**Figure 2:**
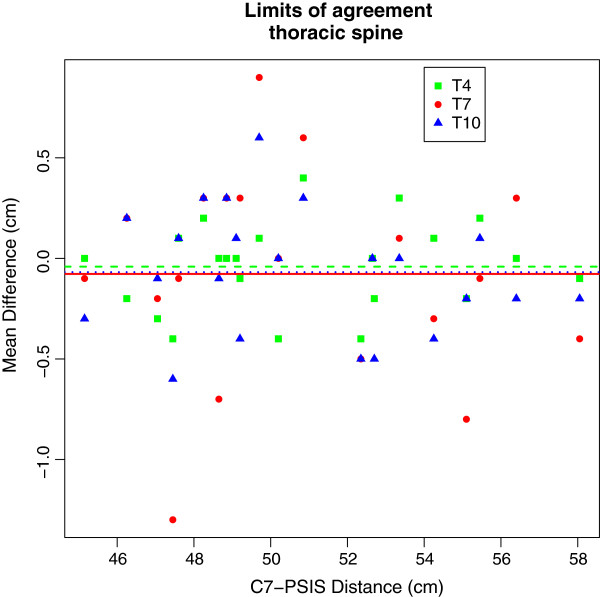
Mean Differences are represented as horizontal lines, individual differences as symbols with values for spinal process T4 in green, T7 in red and T10 in blue.

**Figure 3:**
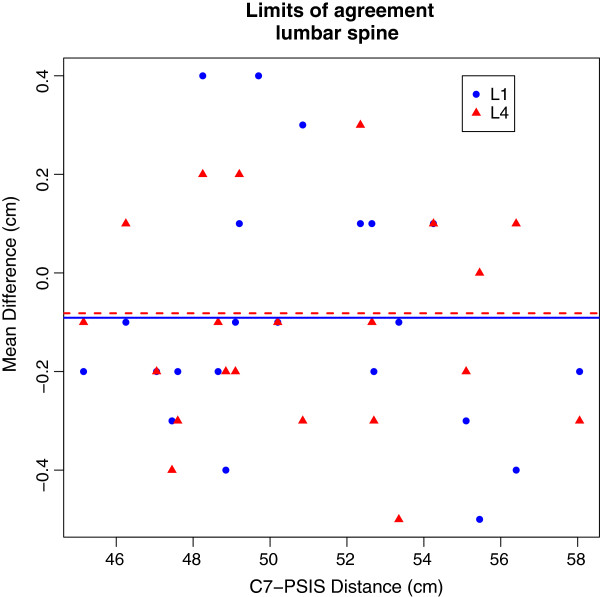
Mean Differences are represented as horizontal lines, individual differences as symbols with values for spinal process L1 in blue and L4 in red.

Subjects had a mean age of 34 years (range: 22 to 51 years), and a mean body height of 175 cm with a range between 157 and 197 cm. The mean C7 to PSIS level distance was 50.9 cm (SD: 3.5 cm). The mean C7 to PSIS level distance for women was 49.0 cm (SD: 1.5 cm) that for men 53.7 cm (SD: 2.4 cm). Table 1 shows the tester reliability with values of absolute (LA and SEM) and relative reliability (ICC) Both ICC and 95% CI of ICC values were greater than 0.9 which indicates an almost perfect reliability according to Landis and Koch. The Limits of agreement indicated that there was no bias along the length between C7 and PSIS, although slightly higher values were obtained for the thoracic compared to the lumbar spine (see Figures 2 and 3). With a highest value of 0.3 cm at T7, the SEM can be considered low.

**Table 1:** Intertester-reliability of measuring length and position variables

**Variable**	**ICC, 1,1**	**95% CI of ICC**	**Mean difference (cm)**	**95%LA (cm)**	**SEM (cm)**
**C7-PSIS distance**	0.991	0.978 to 0.996	< 0.1	−0.5 to 0.6	0.2
**T4**	0.967	0.922 to 0.986	<0.1	−0.4 to 0.5	0.2
**T7**	0.981	0.955 to 0.992	0.1	−0.9 to 1.0	0.3
**T10**	0.988	0.972 to 0.995	0.1	−0.6 to 0.7	0.2
**L1**	0.99	0.976 to 0.996	0.1	−0.4 to 0.6	0.2
**L4**	0.999	0.998 to 1	0.1	−0.4 to 0.6	0.2

Table 1 (ICC = Intraclass Correlation Coefficient, 95% CI = 95% Confidence Interval, 95% LA = 95% Limits of agreement), SEM = Standard error of the measurement, C7-PSIS distance = distance between spinal process of the seventh cervical vertebrae and the posterior superior iliac spine, T4, T7, T10, L1 and L4 = spinal process of the fourth, seventh and tenth thoracic and first and fourth lumbar vertebrae.

Table 2 shows mean percentage estimates and the 95% confidence intervals around the mean percentage value, for the five spinal processes measured. As an example, the T4 position was located on average at 21% of the distance between C7 and PSIS level. Mean percentage estimates of the five spinal processes differed slightly between men and women; however the differences are small with a maximum difference of 1.8%.

**Table 2:** Percentage position on the distance C7 to PSIS

**Variable**	**Value**	**Lower limit**	**Upper limit**
**C7-PSIS**	100%		
**T4**	21.0%	20.0%	21.9%
**T7**	39.0%	38.0%	40.0%
**T10**	54.1%	52.8%	55.4%
**L1**	70.9%	69.5%	72.3%
**L4**	86.1%	85.0%	87.2%

Table 2 (values are means, limits are 95% confidence limits according to a t-distribution with 21 degrees of freedom), C7-PSIS = distance between spinal process of the seventh cervical vertebrae and the posterior superior iliac spine, T4, T7, T10, L1 and L4 = spinal process of the fourth, seventh and tenth thoracic, and first and fourth lumbar vertebrae.

## Discussion

The main result of this study is that, using a percentage model marker placement improves reliability and comparability of our selected landmarks between and within measured subjects in the movement lab. This is true in individuals with no apparent deformation in the sagittal and frontal plane and for upright standing postures.

The thoracic spine length, including L1, of 36 cm and lumbar spine length to the PSIS level of 14.8 cm compared favourably with that reported by Dunleavy *et al*., with values of 32 cm for the thoracic and 12.4 cm for the lumbar spine [14]. Differences might be explained by unequal test protocols. Dunleavy and colleagues measured between C7 and S1. They determined the thoracic and lumbar length by the end of the curve and not by a vertebra [14].

Intertester reliability was found to be almost perfect and is generally poorer than Intratester reliability in manual palpation studies [6,21]. We determined ICC values arising from different tester pairs, although some pairs only occurred once. We used ICC Model 1, 1 to assess reliability of measurements. According to Shrout & Fleiss this model is appropriate to assess the reliability if each subject is measured once by different tester pairs and the testers are selected at random [25]. The error arising from this ICC model is by tester error or from subjects changing their positions slightly between measurements. As measurements were conducted in immediate succession, and no postural changes occurred, we could limit the measurement error considerably. However, within-subject variability might change between days and measurements during the same day or if the subject is measured in a different standing position. However we didn’t examine the reliability of palpatory accuracy, but the reliability of measuring markings on the skin using a flexible ruler. These results indicate that our approach is both reliable and allows fast identification of specific vertebrae in a laboratory setting.

Palpatory accuracy can be confirmed by imaging techniques such as x-ray, MRI or Ultrasound and may have enhanced the validity of our results. We dismissed x-ray imaging on ethical grounds, MRI as it does not image bone tissue and ultrasound as it does not allow the entire spine to be imaged accurately. We also dismissed retrospective evaluation of x-ray images taken of patients in a standing position, as we considered the inherent inaccuracies in this approach to be too great.

Although our approach has been shown to be very accurate, its validity remains to be demonstrated. In order to demonstrate its validity we would have had to perform x-ray imaging, which given that all our participants were healthy, was considered unethical. In order to improve the validity of our palpation procedure, we always used two testers, used functional criteria and anatomical features to identify a particular vertebra [15,17,19,20,22,23]. By using a functional approach, the accuracy of correctly identifying C7 increased from 37.5% to 77.1% in a study by anaesthesiologists [22]. Using x-ray images, Kim et al. validated the position of PSIS level to S1 or S1-S2 interspace level in 73% of examined cases [19]. It has to be considered though, that the authors had their subjects lay in a prone position with a pillow under the abdomen. This may have caused the sacrum to rotate posteriorly in relation to the innominate, altering the relative position of defined landmarks.

Teoh and colleagues identified T7 by counting down vertebrae starting at C7. By comparing the identified vertebrae with x-ray images of the spine, they found correct identification of T7 in 29% of the cases and in nearly half of the errors involved identification of adjacent T6 or T8 [16]. A possible explanation for the error rates might be that the authors did not confirm the right C7 level by cervical extension, and only one tester made the palpation [16]. Another source of error is the assumption that T7 is located at the height of the lower tip of the scapula. The vertebra more closely associated with the lower tip of the scapula is T9 [16,17]; which corresponds to our observation.

Combining anatomical features contributes to improving accuracy and validity of identifying a particular vertebra compared to using a single anatomical feature only, as shown for L4 vertebra [19,20]. Both studies found that testers most likely determine 1 or 2 spinal levels above the correct one, when using the iliac crest as the only reference.

To identify L4, we counted vertebrae up starting at PSIS level and used the shape of the spinal process to differentiate L4 from L5. As a final check we counted both downwards starting at L1 and compared the height to iliac crest level [24].

There are some issues relating to the validity of our approach, as there is some variability in body composition in general and in C7 to PSIS level distance in particular. In our sample, the percentage standard deviations for T4 were between 2.07% and 3.16% for L1 position. Given that in 95% of the cases the correct spinal process is within ± 2 SD, a variation of about 8% is expected for T4 and nearly 13% for L1. There are 20 vertebral segments located between C7 and PSIS level. The average vertebral segment therefore represents 5% of the total distance between C7 and S2. In spite of the reported variability, lumbar vertebrae have higher endplates and intervertebral discs [28,29], we correctly identified the proper spinal process to within two spinal processes for T4 and to within three spinal processes for L1, using our percentage method. This is as accurate as the values reported in most palpation studies [9,16,19,20,22-24].

Variations in the vertebral form may compromise the accuracy of our procedure. A recent study by Snider et al. has found no additional vertebrae and only 3 out of 60 LBP patients exhibited a sacralisation of L5 [24]. We observed no case where an additional vertebra was present or a vertebra was missing. Miscounting seems highly unlikely as an explanation for this, although a lumbarisation or sacralisation can’t be excluded. In future studies our results should be confirmed using image –guided criterion such as x-rays with opaque markers on the skin locate above spinal processes. The exact location on the spinal process should be determined in terms of its upper and lower boundaries, as we assume a spinal process can be as long as vertebral body height, which in turn has been measured between 14 and 23 mm in the thoracic, and 23 and 24 mm in the lumbar spine in mean [28,29].

Our model for placing external marker or sensors on estimated locations makes measurements within and between individuals reliable, and thus comparable, while at the same time reducing the time required to identify a particular vertebra in a laboratory setting.

## Conclusions

A new model for marker and sensor placement for three thoracic (T4, T7 and T10) and two lumbar vertebrae (L1 and L4) in kinematic movement labs has been presented. Testers in the lab can reliably place markers or sensors using percentage positions of the distance between C7 and the PSIS level of the spine, by means of a flexible ruler. Percentage positions correspond to aforementioned spinal processes for subjects without visible deformities in the sagittal and frontal plane. Using this model makes marker or sensor placement reliable, comparable and feasible. Nevertheless the accuracy of the manual palpation method used has to be confirmed subsequently by image-guided procedures.

## Competing interests

The authors declare that they have no competing interests.

## Authors’ contributions

MJE developed the measurement protocol, carried out measurements analysed the data and drafted the manuscript. FMR developed the measurement protocol, carried out measurements and revised the manuscript. CMB developed the measurement protocol, carried out measurements and revised the manuscript. VLM revised and corrected the manuscript, including language corrections. JK developed the measurement protocol, drafted and revised the manuscript. All authors read and approved the final manuscript.

## Supplementary Material

Additional file 1 Raw data is given in centimeters unless stated otherwise. Click here for file
